# Supplementary material for: Comparison of Clinical Outcomes after Non-ST-Segment and ST-Segment Elevation Myocardial Infarction in Diabetic and Nondiabetic Populations
Source: J Clin Med. 2022 Aug 29;11(17):5079. doi: 10.3390/jcm11175079 (PMC9456669; doi:10.3390/jcm11175079)
Supplement: Supplementary file 1 [file jcm-11-05079-s001.zip › jcm-1862871-supplementary.pdf]

Comparison of clinical outcomes after non-ST-segment and ST-segment elevation myocardial infarction in diabetic and nondiabetic populations

Running title: STEMI versus NSTEMI in DM/non-DM

Yong Hoon Kim[a,*,1], Ae-Young Her[a,1], Seung-Woon Rha[b,*], Cheol Ung Choi[b], Byoung Geol Choi[c], Ji Bak Kim[b], Soohyung Park[b], Dong Oh Kang[b], Ji Young Park[d], Sang-Ho Park[e], Myung Ho Jeong[f]

Supplementary Appendix

Supplementary Table S1 Results of collinearity test for MACE in patients with DM or non-DM

Supplementary Table S2 Baseline characteristics of the NSTEMI and STEMI groups before and after PSM analysis

Supplementary Table S3 Baseline clinical, laboratory, angiographic, and procedural characteristics of the NSTEMI and STEMI groups

Supplementary Table S4 Causes of non-cardiac death

Supplementary Table S5 Independent predictors for MACE

Supplementary Table S6 Results of the collinearity test for MACE between the NSTEMI and STEMI groups

Supplementary Figure S1 Subgroup analysis for MACE in patients with diabetes (A) and non-diabetes (B)

Supplementary Table S1. Results of collinearity test for MACE in patients with DM or non-DM

	Variance Inflation Factors	Tolerance	Condition Index
Male	1.372	0.729	1.000
Age	2.098	0.477	3.783
LVEF	1.260	0.793	3.836
BMI	1.286	0.777	4.026
SBP	3.309	0.302	4.178
DBP	3.142	0.318	4.271
Cardiogenic shock	1.313	0.761	4.606
Killip class I/II	1.173	0.853	4.678
CPR on admission	1.083	0.923	4.826
Hypertension	1.197	0.835	4.961
Dyslipidemia	1.047	0.955	5.114
Previous MI	1.324	0.755	5.153
Previous PCI	1.339	0.747	5.204
Previous CABG	1.031	0.970	5.226
Previous HF	1.027	0.974	5.280
Previous CVA	1.041	0.960	5.336
Current smoker	1.365	0.733	5.337
Peak CK-MB	1.182	0.846	5.375
Peak troponin-I	1.093	0.915	5.397
NT-ProBNP	1.190	0.840	5.435
Serum creatinine	1.204	0.831	5.475
eGFR	1.936	0.517	5.564
Total cholesterol	2.600	0.385	5.665
Triglyceride	1.233	0.811	5.708
LDL-cholesterol	2.342	0.427	5.768
Clopidogrel	2.951	0.339	5.893
Ticagrelor	2.549	0.392	6.126
Prasugrel	1.908	0.524	6.536
ACEI	1.921	0.521	6.951
ARB	1.862	0.537	7.102
BB	1.165	0.859	7.127
CCB	1.071	0.933	7.381
Lipid lowering agent	1.124	0.890	8.047
Year of index MI	1.574	0.635	8.417
Left main (IRA)	8.362	0.120	8.817
LAD (IRA)	93.003	0.011	9.068
LCx (IRA)	53.660	0.019	10.153
RCA (IRA)	85.238	0.012	11.090
Left main (treated vessel)	2.320	0.431	11.307
LAD (treated vessel)	3.896	0.257	11.686
LCx (treated vessel)	2.860	0.350	11.929
RCA (treated vessel)	4.856	0.206	12.855

ACC/AHA Type B2 lesion	1.759	0.569	14.182
ACC/AHA Type C lesion	1.850	0.541	14.462
1-vessel disease	1.390	0.834	14.549
2-vessel disease	1.417	0.706	15.043
≥ 3-vessel disease	1.583	0.632	15.501
Pre-PCI TIMI 0/1	1.170	0.855	15.962
PCI within 24 hours	1.044	0.958	17.165
GP IIb/IIIa inhibitor	1.087	0.920	19.476
Transradial approach	1.113	0.898	21.871
IVUS	1.085	0.921	23.211
OCT	1.028	0.973	27.415
FFR	1.031	0.969	31.283
ZES	6.033	0.166	38.857
EES	6.581	0.152	40.408
BES	3.684	0.271	46.372
Stent diameter	1.202	0.832	60.309
Stent length	1.351	0.740	70.270
Number of stent	2.142	0.467	162.182

MACE, major adverse cardiac events; LVEF, left ventricular ejection fraction; BMI, body mass index; SBP, systolic blood pressure; DBP, diastolic blood pressure; CPR, cardiopulmonary resuscitation; PCI, percutaneous coronary intervention; CABG, coronary artery bypass graft; HF, heart failure; CVA, cerebrovascular accidents; CK-MB, creatine kinase myocardial band; NT-ProBNP, N-terminal pro-brain natriuretic peptide; eGFR, estimated glomerular filtration rate; LDL, low density lipoprotein; ACEI, angiotensin converting enzyme inhibitor; ARB, angiotensin receptor blocker; BB, beta-blocker; CCB, calcium channel blocker; IRA, infarct-related artery; LAD, left anterior descending coronary artery; LCx, left circumflex coronary artery; RCA, right coronary artery; ACC/AHA American College of Cardiology/American Heart Association; TIMI, thrombolysis in myocardial infarction; GP, glycoprotein; IVUS, intravascular ultrasound; OCT, optical coherence tomography; FFR, fractional flow reserve; ZES, zotarolimus-eluting stent; EES, everolimus-eluting stent; BES, biolimus-eluting stent.

Supplementary Table S2. Baseline characteristics of the NSTEMI and STEMI groups before and after PSM analysis

Variables	Total (n = 11,798)				PSM patients (n = 5768)			
	NSTEMI (n = 5093)	STEMI (n = 6705)	p value	SD	NSTEMI (n = 2884)	STEMI (n = 2884)	p value	SD
Male, n (%)	3626 (71.2)	5184 (77.3)	<0.001	-1.40	2125 (73.7)	2117 (73.4)	0.811	0.07
Age, years	64.4 ± 12.0	62.1 ± 12.5	<0.001	1.88	63.4 ± 12.3	63.3 ± 12.7	0.816	0.09
LVEF, %	54.1 ± 11.1	50.7 ± 11.0	<0.001	3.10	52.7 ± 11.2	52.5 ± 11.0	0.649	0.04
BMI, kg/m^2	24.1 ± 3.2	24.2 ± 3.1	0.076	-0.32	24.1 ± 3.1	24.2 ± 3.2	0.823	-0.03
SBP, mmHg	134.8 ± 26.6	127.7 ± 27.9	<0.001	2.60	131.1 ± 26.3	131.6 ± 27.3	0.472	-0.19
DBP, mmHg	80.7 ± 15.2	78.4 ± 16.8	<0.001	1.00	79.4 ± 15.7	79.6 ± 16.1	0.564	-0.13
Cardiogenic shock, *n* (%)	113 (2.2)	395 (5.9)	<0.010	-1.89	93 (3.2)	90 (3.1)	0.881	0.06
Killip class I/II, *n* (%)	4440 (87.2)	5613 (83.7)	<0.001	0.99	2484 (86.1)	2483 (86.1)	0.970	0.03
CPR on admission, *n* (%)	122 (2.4)	374 (5.6)	<0.001	-1.63	89 (3.1)	91 (3.2)	0.940	-0.06
Hypertension, *n* (%)	2779 (54.6)	3120 (46.5)	<0.001	1.62	1456 (50.5)	1467 (50.9)	0.772	-0.08
Dyslipidemia, *n* (%)	661 (13.0)	736 (11.0)	0.001	0.62	335 (11.6)	337 (11.7)	0.935	-0.03
Previous MI, *n* (%)	234 (4.6)	196 (2.9)	<0.001	0.90	103 (3.6)	107 (3.7)	0.779	-0.05
Previous PCI, *n* (%)	386 (7.6)	297 (4.4)	<0.001	1.35	156 (5.4)	158 (5.5)	0.908	-0.04
Previous CABG, *n* (%)	31 (0.6)	20 (0.3)	0.015	0.45	11 (0.4)	13 (0.5)	0.838	-0.15
Previous HF, *n* (%)	72 (1.4)	55 (0.8)	0.002	0.58	39 (1.4)	31 (1.1)	0.400	0.27
Previous CVA, *n* (%)	387 (7.6)	326 (4.9)	<0.001	1.12	154 (5.3)	149 (5.2)	0.813	0.05
Current smokers, *n* (%)	1922 (37.7)	3194 (47.6)	<0.001	-2.01	1221 (42.3)	1241 (43.0)	0.613	-0.14
Peak CK-MB, mg/dL	23 (7.0-82.9)	124.0 (34.6-268.5)	<0.001	-6.23	36.4 (9.0-119.6)	42.8 (11.5-124.5)	0.061	-0.40
Peak Troponin-I, ng/mL	11.0 (2.2-47.8)	46.8 (16.0-61.2)	<0.001	-4.66	18.6 (3.0-48.0)	23.2 (4.5-49.4)	0.066	-0.53
NT-ProBNP, pg/mL	658.0 (160.0-2740.5)	265.0 (59.0-1443.0)	<0.001	1.81	483 (134.0-1907.3)	462 (125.4-1802.1)	0.719	0.09
Hs-CRP, mg/dL	10.1 ± 46.8	9.4 ± 37.9	0.427	0.16	10.1 ± 39.1	10.7 ± 40.8	0.579	-0.15
Serum creatinine, mg/dL	1.17 ± 1.70	1.06 ± 1.20	<0.001	0.75	1.09 ± 1.07	1.05 ± 1.00	0.168	0.38

eGFR, mL/min/1.73m^2	88.3 ± 45.3	87.3 ± 37.2	0.215	0.24	89.9 ± 45.2	88.3 ± 39.3	0.121	0.37
Total cholesterol, mg/dL	181.8 ± 46.3	184.0 ± 43.7	0.013	-0.49	183.8 ± 47.4	184.3 ± 44.8	0.726	-1.00
Triglyceride, mg/L	136.3 ± 117.5	137.9 ± 109.8	0.445	-0.14	137.2 ± 123.3	137.5 ± 111.5	0.927	-0.03
HDL cholesterol, mg/L	43.0 ± 14.1	43.3 ± 15.4	0.291	-0.20	43.2 ± 15.3	43.3 ± 15.3	0.853	-0.06
LDL cholesterol, mg/L	114.7 ± 41.7	115.9 ± 39.5	0.096	-0.30	115.3 ± 37.5	115.9 ± 38.4	0.510	-0.16
Discharge medications								
Aspirin, *n* (%)	4940 (97.2)	6484 (96.7)	0.370	0.29	2795 (96.9)	2792 (96.8)	0.821	0.06
Clopidogrel, *n* (%)	4325 (84.9)	5780 (86.2)	0.049	-0.37	2500 (86.7)	2494 (86.5)	0.817	0.06
Ticagrelor, *n* (%)	484 (9.5)	607 (9.1)	0.403	0.14	244 (8.5)	239 (8.3)	0.849	0.07
Prasugrel, *n* (%)	236 (4.6)	366 (5.5)	0.047	-0.41	129 (4.5)	134 (4.6)	0.753	-0.05
Cilostazol, *n* (%)	887 (17.4)	1242 (18.5)	0.122	-0.29	521 (18.1)	523 (18.1)	0.973	-0.03
ACEIs, *n* (%)	2581 (50.7)	3849 (57.4)	<0.001	-1.35	1584 (54.9)	1569 (54.4)	0.692	1.00
ARBs, *n* (%)	1558 (30.6)	1576 (23.5)	<0.001	1.60	726 (25.2)	757 (26.2)	0.366	-0.23
BBs, *n* (%)	4219 (82.8)	5641 (84.1)	0.061	-0.35	2394 (83.0)	2388 (82.8)	0.834	0.05
CCBs, *n* (%)	501 (9.8)	243 (3.6)	<0.001	2.50	178 (6.2)	181 (6.3)	0.913	-0.04
Lipid lowering agents, *n* (%)	4427 (86.9)	5746 (85.7)	0.056	0.35	2469 (85.6)	2477 (85.9)	0.763	-0.09
IRA								
Left main, *n* (%)	127 (2.5)	75 (1.1)	<0.001	1.05	45 (1.6)	44 (1.5)	0.915	0.08
LAD, *n* (%)	2178 (42.8)	3548 (52.9)	<0.001	-2.03	1380 (47.9)	1395 (48.4)	0.712	-1.00
LCx, *n* (%)	1364 (26.8)	592 (8.8)	<0.001	4.84	460 (16.0)	458 (15.9)	0.971	0.03
RCA, *n* (%)	1424 (28.0)	2490 (37.1)	<0.001	-1.95	1001 (34.7)	983 (34.1)	0.637	0.13
Treated vessel								
Left main, *n* (%)	214 (4.2)	115 (1.7)	<0.001	1.48	72 (2.5)	70 (2.4)	0.932	0.06
LAD, *n* (%)	2888 (56.7)	4035 (60.2)	<0.001	-0.71	1667 (57.8)	1689 (58.6)	0.575	-1.06
LCx, *n* (%)	2016 (39.6)	1076 (16.0)	<0.001	5.46	751 (26.0)	760 (26.4)	0.811	-0.09
RCA, *n* (%)	1868 (36.7)	2824 (42.1)	<0.001	-1.11	1214 (42.1)	1172 (40.6)	0.273	0.30
ACC/AHA lesion type								
Type B1, *n* (%)	703 (13.8)	857 (12.8)	0.105	0.29	402 (13.9)	413 (14.3)	0.705	-0.11

Type B2, *n* (%)	1797 (35.3)	2031 (30.3)	<0.001	1.07	911 (31.6)	919 (31.9)	0.843	-0.06
Type C, *n* (%)	2124 (41.7)	3139 (46.8)	<0.001	-1.03	1277 (44.3)	1259 (43.7)	0.652	0.12
Extent of CAD								
1-vessel, *n* (%)	2208 (43.4)	3551 (53.0)	<0.001	-1.93	1368 (47.4)	1372 (47.6)	0.916	-0.04
2-vessel, n (%)	1724 (33.9)	2010 (30.0)	<0.001	0.84	917 (31.8)	914 (31.7)	0.955	0.02
≥ 3-vessel, *n* (%)	1161 (22.8)	1144 (17.1)	<0.001	1.43	599 (20.8)	595 (20.6)	0.922	0.05
Pre-PCI TIMI 0/1, *n* (%)	2047 (40.2)	4781 (71.3)	<0.001	-6.59	1562 (54.2)	1529 (53.0)	0.398	0.24
PCI within 24 hours, *n* (%)	4396 (86.3)	6477 (96.6)	<0.001	-3.74	2699 (93.6)	2708 (93.9)	0.625	-0.12
GP IIb/IIIa inhibitor, n (%)	480 (9.4)	1458 (21.7)	<0.001	-3.44	388 (13.5)	397 (13.8)	0.759	-0.90
Transradial approach, *n* (%)	1833 (36.0)	1218 (18.2)	<0.001	4.09	731 (25.3)	730 (25.3)	0.976	0.02
IVUS, *n* (%)	1241 (24.4)	1352 (20.2)	<0.001	1.01	653 (22.6)	649 (22.5)	0.900	0.02
OCT, *n* (%)	67 (1.3)	22 (0.3)	<0.001	1.12	26 (0.9)	20 (0.7)	0.460	0.22
FFR, *n* (%)	75 (1.5)	61 (0.9)	0.448	0.55	38 (1.3)	29 (1.0)	0.326	0.28
Types of DES[a]								
ZES, *n* (%)	1647 (32.3)	2372 (35.4)	0.206	-0.66	992 (34.0)	976 (33.8)	0.677	0.04
EES, *n* (%)	2710 (53.2)	3374 (50.3)	0.193	0.58	1493 (51.8)	1494 (51.8)	0.979	0.02
BES, *n* (%)	783 (15.4)	873 (13.0)	0.078	0.69	383 (13.3)	401 (13.9)	0.514	-0.18
Others, *n* (%)	124 (2.4)	190 (2.8)	0.710	-0.25	72 (2.5)	69 (2.4)	0.757	0.06
Stent diameter, mm	3.07 ± 0.42	3.18 ± 0.42	<0.001	-2.62	3.12 ± 0.43	3.11 ± 0.41	0.327	0.24
Stent length, mm	27.8 ± 12.8	26.7 ± 10.2	0.019	0.95	27.2 ± 11.6	27.1 ± 11.1	0.762	0.09
Number of stent	1.61 ± 0.89	1.40 ± 0.70	<0.001	2.62	1.51 ± 0.81	1.51 ± 0.79	0.900	0.07

Values are means ± standard deviation or median (interquartile range) or numbers and percentages. The *p* values for continuous data were obtained from the unpaired t-test. The *p* values for categorical data were obtained from the chi-square or Fisher's exact test. PSM, propensity score matched; NSTEMI, non-ST-segment elevation myocardial infarction; STEMI, ST-segment elevation myocardial infarction; SD, standardized mean difference; LVEF, left ventricular ejection fraction; BMI, body mass index; SBP, systolic blood pressure; DBP, diastolic blood pressure; CPR, cardiopulmonary resuscitation; PCI, percutaneous coronary intervention; CABG, coronary artery bypass graft; HF, heart failure; CVA, cerebrovascular accidents; eGFR, estimated glomerular filtration rate; CK-MB, creatine kinase myocardial band; NT-ProBNP, N-terminal pro-brain natriuretic peptide; Hs-CRP, high sensitivity-C-reactive protein; HDL, high-density lipoprotein; LDL, low-density lipoprotein; ACEIs, angiotensin converting enzyme inhibitors; ARBs, angiotensin receptor blockers; BBs, beta-blockers; CCBs, calcium channel blockers; LAD, left anterior descending coronary artery; LCx, left circumflex coronary artery; RCA, right coronary artery; ACC/AHA, American College of Cardiology/American Heart Association; CAD, coronary artery

disease; TIMI, thrombolysis in myocardial infarction; ZES, zotarolimus-eluting stent; EES, everolimus-eluting stent; BES, biolimus-eluting stent; GP, glycoprotein; IVUS, intravascular ultrasound; OCT, optical coherence tomography; FFR, fractional flow reserve; [a]Drug-eluting stents were composed of ZES (Resolute Integrity stent; Medtronic, Inc., Minneapolis, MN), EES (Xience Prime stent, Abbott Vascular, Santa Clara, CA; or Promus Element stent, Boston Scientific, Natick, MA), and BES (BioMatrix Flex stent, Biosensors International, Morges, Switzerland; or Nobori stent, Terumo Corporation, Tokyo, Japan).

Supplementary Table S3. Baseline clinical, laboratory, angiographic, and procedural characteristics of the NSTEMI and STEMI groups

Variables	Overall (n = 11,798)			NSTEMI (n = 5093)			STEMI (n = 6705)		
	DM (n = 5092)	Non-DM (n = 6706)	p value	DM (n = 2399)	Non-DM (n = 2694)	p value	DM (n = 2693)	Non-DM (n = 4012)	p value
Male, n (%)	3581 (70.3)	5229 (78.0)	<0.001	1585 (66.1)	2041 (75.8)	<0.001	1996 (74.1)	3188 (79.5)	<0.001
Age, years	64.2 ± 11.6	62.3 ± 12.8	<0.001	65.5 ± 11.3	63.5 ± 12.6	<0.001	63.0 ± 11.9	61.4 ± 13.0	<0.001
LVEF, %	51.2 ± 11.6	52.9 ± 10.8	<0.001	52.6 ± 11.9	55.4 ± 10.2	<0.001	50.0 ± 11.1	51.2 ± 10.8	<0.001
BMI, kg/m^2	24.4 ± 3.1	24.0 ± 3.1	<0.001	24.3 ± 3.1	24.0 ± 3.2	0.002	24.5 ± 3.1	24.0 ± 3.1	<0.001
SBP, mmHg	131.1 ± 27.9	130.5 ± 27.4	0.245	134.5 ± 26.8	135.0 ± 26.5	0.493	128.1 ± 28.6	127.5 ± 27.5	0.398
DBP, mmHg	78.8 ± 16.1	79.8 ± 16.4	<0.001	79.8 ± 15.1	81.5 ± 15.7	<0.001	77.8 ± 17.0	78.7 ± 16.7	0.034
Cardiogenic shock, n (%)	237 (4.7)	271 (4.0)	0.104	61 (2.5)	52 (1.9)	0.153	176 (6.5)	219 (5.5)	0.072
Killip class I/II, n (%)	4224 (83.0)	5829 (86.9)	<0.001	2013 (83.9)	2427 (90.1)	<0.001	2211 (82.1)	3402 (84.8)	0.003
CPR on admission, n (%)	188 (3.7)	308 (4.6)	0.016	60 (2.5)	62 (2.3)	0.647	128 (4.8)	246 (6.1)	0.017
Hypertension, n (%)	3072 (60.3)	2827 (42.2)	<0.001	1540 (64.2)	1239 (46.0)	<0.001	1532 (56.9)	1588 (39.6)	<0.001
Dyslipidemia, n (%)	731 (14.4)	666 (9.9)	<0.001	375 (15.6)	286 (10.6)	<0.001	356 (13.2)	380 (9.5)	<0.001
Previous MI, n (%)	247 (4.9)	183 (2.7)	<0.001	149 (6.2)	85 (3.2)	<0.001	98 (3.6)	98 (2.4)	0.005
Previous PCI, n (%)	393 (7.7)	290 (4.3)	<0.001	239 (10.0)	147 (5.5)	<0.001	154 (5.7)	143 (3.6)	<0.001
Previous CABG, n (%)	39 (0.8)	12 (0.2)	<0.001	25 (1.0)	6 (0.2)	<0.001	14 (0.5)	6 (0.1)	0.010
Previous HF, n (%)	78 (1.5)	49 (0.7)	<0.001	50 (2.1)	22 (0.8)	<0.001	28 (1.0)	27 (0.7)	0.128
Previous CVA, n (%)	392 (7.7)	321 (4.8)	<0.001	224 (9.3)	163 (6.1)	<0.001	168 (6.2)	158 (3.9)	<0.001
Current smokers, n (%)	1985 (39.0)	3131 (46.7)	<0.001	803 (33.5)	1119 (41.5)	<0.001	1182 (43.9)	2012 (50.1)	<0.001
Peak CK-MB, mg/dL	47.0 (9.4-149.5)	81.5 (16.7-223.0)	<0.001	18.3 (6.0-63.4)	29.5 (8.7-103.1)	<0.001	102.4 (26.9-236.2)	140.5 (39.6-290.8)	<0.001
Peak Troponin-I, ng/mL	25.0 (3.6-47.8)	31.5 (6.3-48.3)	0.579	8.7 (1.7-42.0)	12.7 (2.6-47.8)	0.489	48.1 (18.5-67.8)	47.8 (12.7-52.1)	0.383
Blood glucose, mg/dL	225.9 ± 99.7	142.6 ± 49.0	<0.001	216.5 ± 103.1	130.6 ± 43.4	<0.001	234.3 ± 95.9	150.8 ± 50.8	<0.001
Hemoglobin A1c, %	7.81 ± 2.82	5.67 ± 0.45	<0.001	7.72 ± 2.60	5.67 ± 0.45	<0.001	7.90 ± 3.00	5.67 ± 0.45	<0.001
NT-ProBNP, pg/mL	830.0 (151.0-3617.0)	326.5 (74.0-1428.3)	<0.001	1325.5 (301.0-5417.0)	484.0 (130.5-1857.5)	<0.001	453.5 (89.0-2621.8)	225.0 (53.0-1195.0)	<0.001
Hs-CRP, mg/dL	11.0 ± 43.5	8.7 ± 40.8	0.003	10.7 ± 43.9	9.5 ± 49.2	0.374	11.3 ± 43.1	8.2 ± 33.9	0.001

Serum creatinine, mg/dL	1.21 ± 1.64	1.03 ± 1.27	<0.001	1.34 ± 2.21	1.03 ± 1.06	<0.001	1.10 ± 0.86	1.03 ± 1.39	0.014
eGFR, mL/min/1.73m^2	84.3 ± 43.6	90.3 ± 38.5	<0.001	83.1 ± 46.0	92.8 ± 44.1	<0.001	85.3 ± 41.3	88.6 ± 34.1	0.001
Total cholesterol, mg/dL	179.0 ± 47.8	186.2 ± 42.2	<0.001	177.3 ± 49.8	186.0 ± 42.4	<0.001	180.5 ± 45.8	186.3 ± 42.1	<0.001
Triglyceride, mg/L	150.6 ± 129.9	127.0 ± 97.5	<0.001	150.2 ± 136.2	123.9 ± 96.3	<0.001	151.0 ± 124.0	129.0 ± 98.2	<0.001
HDL cholesterol, mg/L	41.8 ± 14.0	44.2 ± 15.4	<0.001	41.6 ± 13.5	44.2 ± 14.4	<0.001	42.0 ± 14.4	44.1 ± 16.0	<0.001
LDL cholesterol, mg/L	110.6 ± 38.0	119.0 ± 42.0	<0.001	109.3 ± 38.8	119.5 ± 43.6	<0.001	111.8 ± 37.2	118.7 ± 40.8	<0.001
Diabetes management									
Diet, *n* (%)	410 (8.0)			166 (6.9)			244 (9.0)		
Oral agent, *n* (%)	3173 (62.3)			1492 (62.2)			1681 (62.4)		
Insulin, *n* (%)	288 (5.7)			160 (6.7)			128 (4.8)		
Untreated, *n* (%)	1221 (24.0)			581 (24.2)			640 (23.8)		
Discharge medications									
Aspirin, *n* (%)	4922 (96.7)	6502 (97.0)	0.363	2326 (97.0)	2614 (97.0)	0.935	2596 (96.4)	3888 (96.9)	0.250
Clopidogrel, *n* (%)	4450 (87.4)	5655 (84.3)	<0.001	2115 (88.2)	2210 (82.0)	<0.001	2335 (86.7)	3445 (85.9)	0.329
Ticagrelor, *n* (%)	402 (7.9)	689 (10.3)	<0.001	183 (7.6)	301 (11.2)	<0.001	219 (8.1)	388 (9.7)	0.033
Prasugrel, *n* (%)	240 (4.7)	362 (5.4)	0.100	101 (4.2)	135 (4.4)	0.182	139 (5.2)	227 (5.7)	0.411
Cilostazol, *n* (%)	1000 (19.6)	1129 (16.8)	<0.001	462 (19.3)	425 (15.8)	0.001	538 (20.0)	704 (17.5)	0.012
ACEIs, *n* (%)	2588 (50.8)	3842 (57.3)	<0.001	1133 (47.2)	1448 (53.7)	<0.001	1455 (54.0)	2394 (59.7)	<0.001
ARBs, *n* (%)	1540 (30.2)	1594 (23.8)	<0.001	828 (34.5)	730 (27.1)	<0.001	712 (26.4)	864 (21.5)	<0.001
BBs, *n* (%)	4273 (83.9)	5587 (83.3)	0.382	2007 (83.7)	2212 (82.1)	0.143	2266 (84.1)	3375 (84.1)	0.981
CCBs, *n* (%)	380 (7.5)	364 (5.4)	<0.001	265 (11.0)	236 (8.8)	0.007	115 (4.3)	128 (3.2)	0.023
Lipid lowering agents, *n* (%)	4300 (84.4)	5873 (87.6)	<0.001	2038 (85.0)	2389 (88.7)	<0.001	2262 (84.0)	3484 (86.8)	0.001
Year of index MI			<0.001			<0.001			<0.001
Before 2013	3314 (65.1)	4017 (59.9)		1480 (61.7)	1479 (54.9)		1834 (68.1)	2538 (63.3)	
After 2013	1778 (34.9)	2689 (40.1)		919 (38.3)	1215 (45.1)		859 (31.9)	1474 (36.7)	
IRA									
Left main, *n* (%)	97 (1.9)	105 (1.6)	0.173	66 (2.8)	61 (2.3)	0.281	31(1.2)	44 (1.1)	0.906
LAD, *n* (%)	2349 (46.1)	3377 (50.4)	<0.001	1006 (41.9)	1172 (43.5)	0.337	1343 (49.9)	2205 (55.0)	<0.001

LCx, *n* (%)	858 (16.9)	1098 (16.4)	0.500	625 (26.1)	739 (27.4)	0.267	233 (8.7)	359 (8.9)	0.693
RCA, *n* (%)	1788 (35.1)	2126 (31.7)	<0.001	702 (29.3)	722 (26.8)	0.053	1086 (40.3)	1404 (35.0)	<0.001
Treated vessel									
Left main, *n* (%)	149 (2.9)	180 (2.7)	0.430	105 (4.4)	109 (4.0)	0.567	44 (1.6)	71 (1.8)	0.702
LAD, *n* (%)	2960 (58.1)	3963 (59.1)	0.291	1378 (57.4)	1510 (56.1)	0.322	1582 (58.7)	2453 (61.1)	0.049
LCx, *n* (%)	1411 (27.7)	1681 (25.1)	0.001	957 (39.9)	1059 (39.3)	0.672	454 (16.9)	622 (15.5)	0.138
RCA, *n* (%)	2173 (42.7)	2519 (37.6)	<0.001	937 (39.1)	931 (34.6)	0.001	1236 (45.9)	1588 (39.6)	<0.001
ACC/AHA lesion type									
Type B1, *n* (%)	654 (12.8)	906 (13.5)	0.297	327 (13.6)	376 (14.0)	0.736	327 (12.1)	530 (13.2)	0.205
Type B2, *n* (%)	1660 (32.6)	2168 (32.3)	0.756	835 (34.8)	962 (35.7)	0.501	825 (30.6)	1206 (30.1)	0.615
Type C, *n* (%)	2305 (45.3)	2958 (44.1)	0.210	1025 (42.7)	1099 (40.8)	0.163	1280 (47.5)	1859 (46.3)	0.337
Extent of CAD									
1-vessel, *n* (%)	2177 (42.8)	3582 (53.4)	<0.001	910 (37.9)	1298 (48.2)	<0.001	1267 (47.0)	2284 (56.9)	<0.001
2-vessel, *n* (%)	1693 (33.2)	2041 (30.4)	0.001	848 (35.3)	876 (32.6)	0.033	845 (31.4)	1165 (29.0)	0.040
≥ 3-vessel, *n* (%)	1222 (24.0)	1083 (16.1)	<0.001	641 (26.7)	520 (19.3)	<0.001	581 (21.6)	563 (14.0)	<0.001
Pre-PCI TIMI 0/1, *n* (%)	2787 (54.7)	4041 (60.3)	<0.001	903 (37.6)	1144 (42.5)	<0.001	1884 (70.0)	2897 (72.2)	0.046
PCI within 24 hours, *n* (%)	4639 (91.1)	6234 (93.0)	<0.001	2040 (85.0)	2356 (87.5)	0.012	2599 (96.5)	3878 (96.7)	0.739
GP IIb/IIIa inhibitor, *n* (%)	729 (14.3)	1209 (18.0)	<0.001	202 (8.4)	278 (10.3)	0.021	527 (19.6)	931 (23.2)	<0.001
Transradial approach, *n* (%)	1308 (25.7)	1743 (26.0)	0.718	815 (34.0)	1018 (37.8)	0.005	493 (18.3)	725 (18.1)	0.806
IVUS, *n* (%)	1081 (21.2)	1512 (22.5)	0.089	539 (22.5)	702 (26.1)	0.003	542 (20.1)	810 (20.2)	0.975
OCT, *n* (%)	36 (0.7)	53 (0.8)	0.666	26 (1.1)	41 (1.5)	0.178	10 (0.4)	12 (0.3)	0.666
FFR, *n* (%)	62 (1.2)	74 (1.1)	0.603	38 (1.6)	37 (1.4)	0.561	24 (0.9)	37 (0.9)	0.896
Types of DES[a]									
ZES, *n* (%)	1765 (34.7)	2254 (33.6)	0.233	789 (32.9)	858 (31.8)	0.428	976 (36.2)	1396 (34.8)	0.225
EES, *n* (%)	2625 (51.6)	3459 (51.6)	0.975	1289 (53.7)	1421 (52.7)	0.482	1336 (49.6)	2038 (50.8)	0.340
BES, *n* (%)	665 (13.1)	991 (14.8)	0.008	335 (14.0)	448 (16.6)	0.008	330 (12.3)	543 (13.5)	0.129
Others, *n* (%)	161 (3.2)	153 (2.3)	0.004	72 (3.0)	52 (1.9)	0.014	89 (3.3)	101 (2.5)	0.061
Stent diameter, mm	3.10 ± 0.42	3.15 ± 0.42	<0.001	3.15 ± 0.42	3.19 ± 0.41	<0.001	3.04 ± 0.41	3.09 ± 0.42	<0.001

Stent length, mm	27.5 ± 11.7	26.9 ± 11.2	0.015	28.0 ± 12.9	27.6 ± 12.6	0.217	27.0 ± 10.5	26.5 ± 10.0	0.081
Number of stent	1.55 ± 0.81	1.46 ± 0.76	<0.001	1.67 ± 0.90	1.56 ± 0.88	<0.001	1.44 ± 0.74	1.40 ± 0.66	<0.001

Values are means ± standard deviation or median (interquartile range) or numbers and percentages. The p values for continuous data were obtained from the unpaired t-test. The p values for categorical data were obtained from the chi-square or Fisher's exact test. NSTEMI, non-ST-segment elevation myocardial infarction; STEMI, ST-segment elevation myocardial infarction; LVEF, left ventricular ejection fraction; BMI, body mass index; SBP, systolic blood pressure; DBP, diastolic blood pressure; CPR, cardiopulmonary resuscitation; PCI, percutaneous coronary intervention; CABG, coronary artery bypass graft; HF, heart failure; CVA, cerebrovascular accidents; eGFR, estimated glomerular filtration rate; CK-MB, creatine kinase myocardial band; NT-ProBNP, N-terminal pro-brain natriuretic peptide; Hs-CRP, high sensitivity-C-reactive protein; HDL, high-density lipoprotein; LDL, low-density lipoprotein; ACEIs, angiotensin converting enzyme inhibitors; ARBs, angiotensin receptor blockers; BBs, beta-blockers; CCBs, calcium channel blockers; LAD, left anterior descending coronary artery; LCx, left circumflex coronary artery; RCA, right coronary artery; ACC/AHA, American College of Cardiology/American Heart Association; CAD, coronary artery disease; TIMI, thrombolysis in myocardial infarction; ZES, zotarolimus-eluting stent; EES, everolimus-eluting stent; BES, biolimus-eluting stent; GP, glycoprotein; IVUS, intravascular ultrasound; OCT, optical coherence tomography; FFR, fractional flow reserve; [a]Drug-eluting stents were composed of ZES (Resolute Integrity stent; Medtronic, Inc., Minneapolis, MN), EES (Xience Prime stent, Abbott Vascular, Santa Clara, CA; or Promus Element stent, Boston Scientific, Natick, MA), and BES (BioMatrix Flex stent, Biosensors International, Morges, Switzerland; or Nobori stent, Terumo Corporation, Tokyo, Japan).

Supplementary Table S4. Causes of non-cardiac death

	DM (n = 5092)			Non-DM (n = 6706)			Overall (n = 11,798)		
	NSTEMI (n = 2399)	STEMI (n = 2693)	p value	NSTEMI (n = 2694)	STEMI (n = 4012)	p value	NSTEMI (n = 5093)	STEMI (n = 6705)	p value
Non-cardiac death, n (%)	44 (1.8)	21 (0.8)	0.001	18 (0.7)	24 (0.6)	0.753	62 (1.2)	45 (0.7)	0.002
Sepsis, n (%)	3 (0.1)	2 (0.1)	0.672	2 (0.1)	5 (0.1)	0.709	5 (0.1)	7 (0.1)	0.916
Multiple organ failure, n (%)	16 (0.7)	5 (0.2)	0.008	8 (0.3)	10 (0.2)	0.811	24 (0.5)	15 (0.2)	0.023
CVA, n (%)	22 (0.9)	11 (0.4)	0.034	6 (0.2)	5 (0.1)	0.367	28 (0.5)	16 (0.2)	0.009
Bleeding, n (%)	2 (0.1)	2 (0.1)	0.908	1 (0.0)	3 (0.1)	0.653	3 (0.1)	5 (0.1)	0.746
Other, n (%)	1 (0.0)	1 (0.0)	0.935	1 (0.0)	1 (0.0)	0.777	2 (0.0)	2 (0.0)	0.783

	NSTEMI (n = 5093)			STEMI (n = 6705)			Overall (n = 11,798)		
	DM (n = 2399)	Non-DM (n = 2694)	p value	DM (n = 2693)	Non-DM (n = 4012)	p value	Diabetes (n = 5092)	Non-diabetes (n = 6706)	p value
Non-cardiac death, n (%)	44 (1.8)	18 (0.7)	<0.001	21 (0.8)	24 (0.6)	0.446	65 (1.3)	42 (0.6)	<0.001
Sepsis, n (%)	3 (0.1)	2 (0.1)	0.671	2 (0.1)	5 (0.1)	0.709	5 (0.1)	7 (0.1)	0.917
Multiple organ failure, n (%)	16 (0.7)	8 (0.3)	0.042	5 (0.2)	10 (0.2)	0.828	21 (0.4)	18 (0.3)	0.196
CVA, n (%)	22 (0.9)	6 (0.2)	0.001	11 (0.4)	5 (0.1)	0.037	33 (0.6)	11 (0.2)	<0.001
Bleeding, n (%)	2 (0.1)	1 (0.0)	0.605	2 (0.1)	3 (0.1)	0.994	4 (0.1)	4 (0.1)	0.733
Other, n (%)	1 (0.0)	1 (0.0)	0.935	1 (0.0)	1 (0.0)	0.777	2 (0.0)	2 (0.0)	0.782

DM, diabetes mellitus; NSTEMI, non-ST-segment elevation myocardial infarction; STEMI, ST-segment elevation myocardial infarction; CVA, cerebrovascular accidents.

Supplementary Table S5. Independent predictors for MACE

	DM				Non-DM			
	Unadjusted		Adjusted		Unadjusted		Adjusted	
**Variables**	**HR (95% CI)**	***p* value**	**HR (95% CI)**	***p* value**	**HR (95% CI)**	***p* value**	**HR (95% CI)**	***p* value**
NSTEMI vs. STEMI	1.192 (0.986—1.440)	0.069	1.079 (0.853—1.325)	0.525	1.287 (1.052–1.574)	0.014	1.575 (1.225–1.979)	<0.001
Male	1.349 (1.078—1.688)	0.009	1.145 (0.869—1.509)	0.337	1.359 (1.116–1.655)	0.002	1.147 (0.904–1.456)	0.259
Age, ≥65 years	1.505 (1.232—1.840)	<0.001	1.055 (0.825–1.349)	0.671	1.455 (1.200–1.763)	<0.001	1.146 (0.911–1.443)	0.245
LVEF, <40%	2.138 (1.650—2.770)	<0.001	1.534 (1.148–2.050)	0.004	2.536 (2.064–3.117)	<0.001	1.716 (1.364–2.159)	<0.001
BMI	0.940 (0.909—0.971)	0.001	0.972 (0.938–1.006)	0.110	0.979 (0.949–1.010)	0.183	1.013 (0.980–1.046)	0.447
SBP	0.996 (0.993—1.000)	0.062	1.002 (0.998–1.006)	0.415	0.994 (0.991–0.998)	0.002	0.999 (0.995–1.003)	0.521
DBP	0.995 (0.989—1.000)	0.069	1.003 (1.000–1.007)	0.521	0.994 (0.988–1.001)	0.078	1.001 (0.990–1.011)	0.896
Cardiogenic shock	1.827 (1.235—2.701)	0.003	1.637 (1.010–2.653)	0.021	1.641 (1.132–2.378)	0.009	1.841 (1.267–2.541)	0.006
Killip class I/II	1.717 (1.342—2.196)	<0.001	1.070 (0.806–1.420)	0.642	2.080 (1.690–2.558)	<0.001	1.259 (0.994–1.594)	0.059
CPR on admission	3.093 (2.273—4.210)	<0.001	2.612 (1.869–3.651)	<0.001	3.890 (2.865–5.283)	<0.001	2.691 (1.930–3.753)	<0.001
Hypertension	1.209 (0.989—1.477)	0.064	1.021 (0.821–1.271)	0.849	1.228 (1.008–1.497)	0.042	1.007 (0.811–1.251)	0.946
Dyslipidemia	1.035 (0.792—1.352)	0.802	1.007 (0.765–1.326)	0.959	1.030 (0.734–1.446)	0.865	1.125 (0.796–1.589)	0.504
Previous MI	1.019 (0.544—1.909)	0.954	1.069 (0.530–2.157)	0.852	2.476 (1.812–3.383)	<0.001	2.046 (1.427–2.933)	<0.001
Previous PCI	1.012 (0.614—1.670)	0.961	1.145 (0.654–2.007)	0.635	1.468 (1.078–1.999)	0.015	1.029 (0.722–1.467)	0.874
Previous CVA	1.693 (1.166—2.457)	0.006	1.321 (0.893–1.953)	0.163	2.083 (1.591–2.726)	<0.001	1.719 (1.293–2.284)	<0.001
Current smoker	1.222 (0.998—1.497)	0.053	1.021 (0.811–1.287)	0.857	1.269 (1.040–1.550)	0.019	1.103 (0.869–1.400)	0.421
Peak Troponin-I	1.001 (1.000—1.002)	0.040	1.001 (1.000–1.002)	0.019	1.000 (0.999–1.001)	0.019	1.001 (1.000–1.002)	0.010
NT-ProBNP	1.002 (1.001—1.003)	<0.001	1.001 (1.000–1.002)	0.005	1.001 (0.999–1.003)	<0.001	1.001 (0.998–1.002)	0.037
Serum creatinine	1.027 (0.986—1.070)	0.203	1.011 (0.944–1.082)	0.762	1.035 (1.016–1.054)	<0.001	1.072 (1.001–1.148)	0.045
eGFR	0.996 (0.992—0.999)	0.011	0.998 (0.995–1.002)	0.296	0.993 (0.990–0.995)	<0.001	1.000 (0.997–1.003)	0.795
Total cholesterol	0.997 (0.994—0.999)	0.009	0.999 (0.995–1.001)	0.437	0.996 (0.994–0.998)	<0.001	0.999 (0.995–1.003)	0.568
Triglyceride	0.998 (0.997—1.000)	0.008	0.999 (0.998–1.000)	0.411	0.999 (0.998–1.000)	0.027	1.000 (0.999–1.001)	0.974
LDL cholesterol	0.997 (0.995—1.000)	0.068	1.000 (0.997–1.003)	0.800	0.995 (0.993–0.998)	<0.001	0.999 (0.995–1.003)	0.702
Clopidogrel	1.153 (0.829—1.603)	0.398	1.606 (1.124–2.822)	0.100	1.023 (0.768–1.362)	0.876	1.108 (0.824–1.490)	0.497
Ticagrelor	1.273 (0.811—1.996)	0.294	2.123 (0.945–4.513)	0.069	1.001 (0.688–1.456)	0.996	1.581 (0.782–3.193)	0.202

Prasugrel	1.247 (0.745–2.088)	0.401	2.013 (0.938–4.321)	0.073	1.446 (0.832 –2.515)	0.191	1.986 (0.886–4.455)	0.096
ACEI/ARB	1.207 (0.987–1.475)	0.067	1.028 (0.830–1.274)	0.801	1.649 (1.359 –2.001)	<0.001	1.321 (1.072–1.627)	0.009
Beta-blocker	1.584 (1.250–2.005)	<0.001	1.222 (0.944–1.582)	0.128	1.843 (1.482 –2.291)	<0.001	1.294 (1.021–1.640)	0.033
Calcium channel blocker	1.081 (0.765–1.527)	0.659	1.001 (0.697–1.436)	0.998	1.110 (0.700 –1.761)	0.657	1.135 (0.708–1.821)	0.599
Lipid lowering agent	1.915 (1.503–2.440)	<0.001	1.584 (1.224–2.050)	<0.001	1.600 (1.278 –2.003)	<0.001	1.324 (1.041–1.684)	0.022

DM, diabetes mellitus; NSTEMI, non-ST-segment elevation myocardial infarction; STEMI, ST-segment elevation myocardial infarction; HR, hazard ratio; CI, confidence interval; LVEF, left ventricular ejection fraction; BMI, body mass index; SBP, systolic blood pressure; DBP, diastolic blood pressure; CPR, cardiopulmonary resuscitation; PCI, percutaneous coronary intervention; CVA, cerebrovascular accidents; NT-ProBNP, N-terminal pro-brain natriuretic peptide; eGFR, estimated glomerular filtration rate; LDL, low density lipoprotein; ACEI, angiotensin converting enzyme inhibitor; ARB angiotensin receptor blocker.

Supplementary Table S6. Results of the collinearity test for MACE between the NSTEMI and STEMI groups

	Variance Inflation Factors	Tolerance	Condition Index
Male	1.369	0.730	1.000
Age	2.041	0.490	3.328
LVEF	1.212	0.825	3.668
BMI	1.265	0.790	3.998
SBP	3.267	0.306	4.098
DBP	3.125	0.320	4.325
Cardiogenic shock	1.310	0.764	4.502
Killip class I/II	1.165	0.858	4.545
CPR on admission	1.075	0.930	4.670
Hypertension	1.181	0.846	4.700
Previous MI	1.321	0.757	4.829
Previous PCI	1.320	0.744	4.863
Previous CABG	1.027	0.974	5.134
Previous HF	1.024	0.977	5.613
Previous CVA	1.038	0.963	5.913
Current smoker	1.356	0.737	6.042
Peak CK-MB	1.157	0.864	6.231
Peak troponin-I	1.090	0.918	6.375
NT-ProBNP	1.183	0.845	6.537
Serum creatinine	1.199	0.834	7.111
eGFR	1.919	0.521	7.516
Total cholesterol	2.309	0.433	7.967
LDL-cholesterol	2.216	0.451	8.827
Year of index PCI	1.177	0.850	9.641
ACEI	1.911	0.523	10.709
ARB	1.823	0.549	12.272
BB	1.142	0.876	12.523
Lipid lowering agent	1.111	0.900	12.562
Left main (IRA)	2.276	0.439	13.265
Left main (treated vessel)	2.303	0.434	14.127
RCA (treated vessel)	1.230	0.813	14.972
1-vessel disease	1.503	0.665	21.041
≥ 3-vessel disease	1.362	0.734	25.200
Pre-PCI TIMI 0/1	1.108	0.902	31.453
Transradial approach	1.091	0.917	34.269
Stent diameter	1.131	0.884	39.739
Stent length	1.208	0.828	52.234
Number of stent	1.440	0.990	87.700

MACE, major adverse cardiac events; NSTEMI, non-ST-segment elevation myocardial infarction; STEMI, ST-segment elevation myocardial infarction; LVEF, left ventricular ejection fraction; BMI, body mass index; SBP, systolic blood pressure; DBP, diastolic blood

pressure; CPR, cardiopulmonary resuscitation; PCI, percutaneous coronary intervention; CABG, coronary artery bypass graft; HF, heart failure; CVA, cerebrovascular accidents; CK-MB, creatine kinase myocardial band; NT-ProBNP, N-terminal pro-brain natriuretic peptide; eGFR, estimated glomerular filtration rate; LDL, low density lipoprotein; ACEI, angiotensin converting enzyme inhibitor; ARB, angiotensin receptor blocker; BB, beta-blocker; IRA, infarct-related artery; RCA, right coronary artery; TIMI, thrombolysis in myocardial infarction.

A DM

Subgroup	NSTEMI	STEMI	HR (95% CI)	p value	p-for interaction
All patients	219/2399	211/2693	1.19 (0.98-1.44)	0.069	
Sex					0.009
Male	137/1585	138/1996	1.28 (1.05-1.62)	0.037	
Female	82/814	73/697	0.96 (0.70-1.32)	0.833	
Age, years					0.014
< 65	84/1089	93/1448	1.23 (0.92-1.66)	0.159	
≥ 65	135/1310	118/1245	1.10 (0.86-1.41)	0.441	
LVEF, %					<0.001
< 40	72/339	63/435	1.50 (1.07-2.10)	0.018	
≥ 40	147/2060	148/2258	1.11 (0.88-1.40)	0.343	
Cardiogenic shock					0.350
Yes	7/61	23/176	0.85 (0.36-2.00)	0.723	
No	212/2338	188/2517	1.24 (1.02-1.51)	0.027	
CPR on admission					<0.001
Yes	17/60	29/128	1.33 (0.73-2.43)	0.344	
No	202/2339	182/2565	1.25 (1.02-1.52)	0.029	
Hypertension					0.170
Yes	1421540	137/1532	1.04 (0.82-1.31)	0.746	
No	77/859	74/1161	1.46 (1.06-2.02)	0.018	
Dyslipidemia					0.701
Yes	29/375	34/356	0.83 (0.50-1.36)	0.464	
No	190/2024	177/2337	1.26 (1.03-1.55)	0.023	
ACC/AHA type					0.040
A/B1	45/539	39/588	1.31 (0.85-2.01)	0.215	
B2/C	174/1860	172/2105	1.16 (0.94-1.43)	0.155	
Lipid-lowering agent					0.384
Yes	184/2038	164/2262	1.27 (1.03-1.57)	0.023	
No	35/361	47/431	0.90 (0.58-1.04)	0.665	
Mulitvessel disease					0.004
Yes	153/1489	136/1426	1.09 (0.86-1.37)	0.463	
No	66/910	75/1267	1.27 (0.91-1.78)	0.145	
Stent diameter					0.109
< 3.0mm	84/937	67/728	0.98 (0.71-1.35)	0.903	
≥ 3.0mm	135/1462	144/1965	1.29 (1.02-1.64)	0.030	
Stent length					0.011
< 30mm	145/1626	144/1906	1.20 (0.95-1.52)	0.110	
≥ 30mm	74/773	67787	1.14 (0.82-1.59)	0.417	

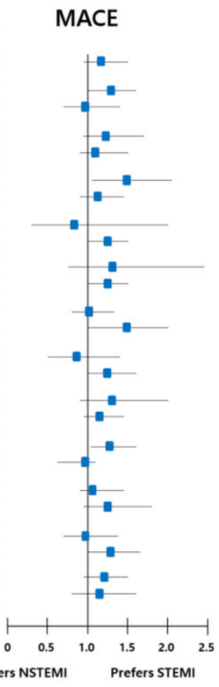

B Non-DM

Subgroup	NSTEMI	STEMI	HR (95% CI)	p value	p-for interaction
All patients	175/2694	208/4012	1.28 (1.05-1.57)	0.014	
Sex					0.007
Male	115/2041	163/3188	1.12 (0.88-1.42)	0.333	
Female	60/653	45/824	1.75 (1.19-2.51)	0.004	
Age, years					0.001
< 65	66/1418	116/2418	0.99 (0.73-1.33)	0.946	
≥ 65	109/1276	92/1594	1.52 (1.15-2.01)	0.003	
LVEF, %					0.002
< 40	26/164	44/481	1.82 (1.12-2.95)	0.015	
≥ 40	149/2530	164/3531	1.30 (1.04-1.62)	0.019	
Cardiogenic shock					0.021
Yes	7/52	20/219	1.47 (0.62-3.48)	0.378	
No	168/2642	188/3793	1.32 (1.07-1.62)	0.009	
CPR on admission					<0.001
Yes	13/62	33/246	1.64 (0.86-3.13)	0.127	
No	162/2632	175/3766	1.36 (1.10-1.69)	0.004	
Hypertension					0.001
Yes	95/1239	84/1588	1.50 (1.12-2.01)	0.007	
No	80/1455	124/2424	1.06 (0.83-1.45)	0.522	
Dyslipidemia					0.226
Yes	21/286	16/380	1.17 (0.92-3.39)	0.085	
No	154/2408	192/3632	1.24 (1.00-1.53)	0.043	
ACC/AHA type					0.001
A/B1	32/633	55/947	0.88 (0.57-1.36)	0.574	
B2/C	143/2061	153/3065	1.43 (1.14-1.80)	0.002	
Lipid-lowering agent					0.213
Yes	145/2389	154/3484	1.41 (1.13-1.78)	0.002	
No	30/305	54/528	0.95 (0.61-1.49)	0.851	
Mulitvessel disease					<0.001
Yes	115/1396	118/728	1.24 (0.96-1.60)	0.096	
No	60/1298	90/2284	1.20 (0.86-1.67)	0.265	
Stent diameter					0.018
< 3.0mm	63/918	50/899	1.26 (0.87-1.83)	0.214	
≥ 3.0mm	112/1776	158/3113	1.27 (1.00-1.62)	0.049	
Stent length					0.013
< 30mm	120/1867	147/2901	1.30 (1.02-1.65)	0.033	
≥ 30mm	55/827	61/1111	1.24 (0.86-1.79)	0.242	

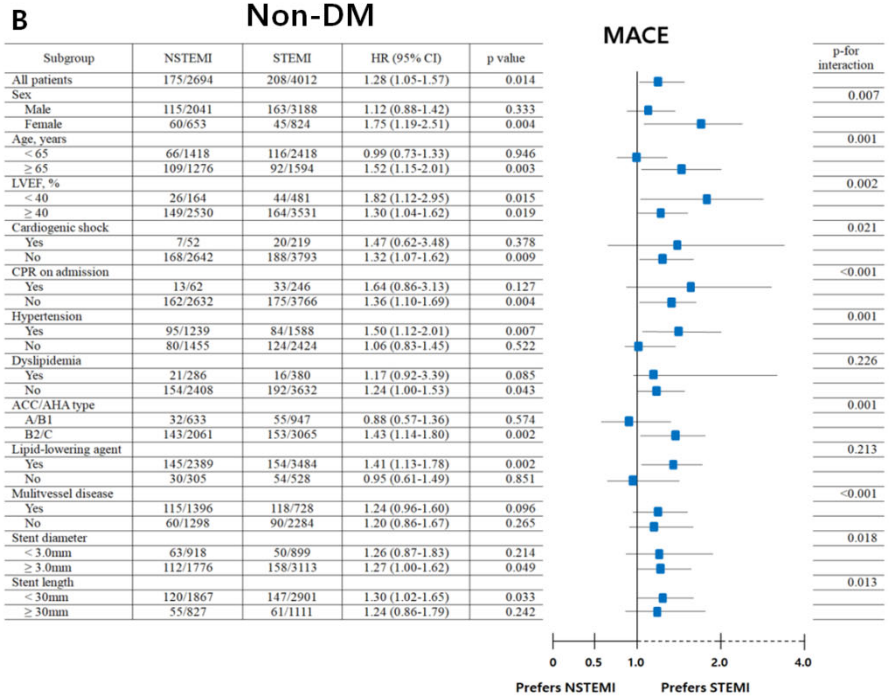

Supplementary Figure S1. Subgroup analysis for MACE in patients with diabetes (A) and non-diabetes (B)
